# Validation of a Questionnaire of Motivations for Moderated and Severe Alcohol Consumption Among College Students

**DOI:** 10.3390/healthcare13030307

**Published:** 2025-02-02

**Authors:** Abel Lerma, Jorge Alberto Soto-Huerta, Cristina J. González-Flores, Rebeca María Elena Guzmán-Saldaña, Diego Aguirre-Villegas, Claudia Lerma

**Affiliations:** 1 Institute of Health Sciences, Universidad Autónoma del Estado de Hidalgo, San Juan Tilcuautla 42160, Mexico or abel.lerma@anahuac.mx (A.L.); rguzman@uaeh.edu.mx (R.M.E.G.-S.); 2 Facultad de Contaduría y Administración de la Universidad Veracruzana, Campus Ixtaczoquitlán, Ixtaczoquitlán 94452, Mexico; joalsohu@hotmail.com; 3 Centro Universitario de la Cienega, University of Guadalajara, Ocotlán 47820, Mexico; cristina.gonzalez4243@academicos.udg.mx; 4 Centro de Investigación en Ciencias de la Salud (CICSA), Facultad de Ciencias de la Salud, Universidad Anahuac Mexico, Huixquilucan 52786, Mexico; daguirre@anahuac.mx; 5 Departamento de Biología Molecular, Instituto Nacional de Cardiologia Ignacio Chávez, Mexico City 04480, Mexico

**Keywords:** alcoholism, college students, motivations, risk factors, questionnaire design

## Abstract

**Objective**: This work aimed to develop and validate a scale to assess motivations for alcohol drinking among Mexican college students. **Methods**: The scale design consisted of applying a stimulus phrase to assess motivations for moderate alcohol drinking (up to three drinks per occasion) and severe alcohol consumption (four or more drinks) in 130 college students. The semantic network technique was applied to identify 15 defining motivations (with more considerable semantic weight) for each drinking level, constituting the pilot scale. The pilot scale was validated on 307 students from a public university in Mexico (255 with moderate drinking and 82 with severe consumption). **Results**: The final number of items per level of drinking was 10 (moderate drinking) and 13 (severe consumption). Internal reliability (Cronbach’s alpha) for the first one was 0.886 with three factors that explain 57.5% of the total variance; the second had an alpha of 0.884 with four factors that explain 70.5% of the total variance. All the factors had positive correlations with the risk perception for alcohol drinking, and there was a positive correlation between severe consumption motivation and the risk perception for consumption of other substances. The confirmatory factor analysis showed that the proposed theoretical models adjust to the data with an error of approximately zero (i.e., RMSEA of 0.088 for moderate consumption and 0.074 for severe consumption), which also carefully measures the motivation for moderate and severe alcohol consumption among college students. **Conclusions**: The new scale is valid and reliable for assessing motivations for moderate and severe alcohol consumption in Mexican college students. This may be a valuable tool to design and evaluate interventions for the prevention of alcohol use among college students.

## 1. Introduction

Alcohol consumption in university settings has become a public health problem that represents a burden in daily life for many university students [1]. Between 49% of university students between 18 and 22 years old consume alcohol, 28.9% do so excessively and are at risk (binge drinking), 40% of them abuse it, and 1 in 12 students meet dependence criteria for alcohol [2]. For many students, alcohol consumption begins at an early age, before university enrollment [3]. In Mexico, according to the National Survey on Drug, Alcohol, and Tobacco Consumption (ENCODAT, 2016), 71% of the Mexican population consumes alcohol, the age range begins at 12 years old, and around 47% of the population between 18 and 25 years old, the highest consumption is achieved, with beer being the most consumed beverage [4,5,6]. Some factors related to alcohol consumption are misinformation, low-risk perception, academic stress, social acceptance, family dysfunction, and the consumption of other substances [7,8,9]. Some consequences of alcohol consumption reach spheres related to the student’s personal and social life. Fights between peers, sexual assaults, low academic performance [9], and alcohol-related injuries are some of the consequences [10,11]. Furthermore, drinking alcohol correlates negatively with well-being [12] and with poorer mental health, since alcohol use is associated with depression [13], anxiety [14], and stress [15].

Different motivations have been identified for alcohol consumption, including increased confidence in social relationships, the search for pleasurable sensations, the sense of belonging in peer groups, the avoidance of rejection, and the relief of negative emotions and personal situations [3,16,17,18,19]. Since motivations precede behavior, assessing the motivations for alcohol consumption is necessary to understand how different drinking motives lead to either reduced alcohol use (i.e., drinking responsibly) or heavy drinking and their subsequent alcohol-related problems [1,20]. These different drinking motives could also be related to different coping styles when facing depression and anxiety symptoms [21], which is essential when implementing coping-based alcohol use interventions.

The growing interest in studying alcohol consumption, its causes, motivations, and risk factors has led to the design of tools to assess consumption. Some questionnaires evaluate drinking motives, such as the Drinking Motives Questionnaire-Revised (DMQR), which consists of 28 items in four dimensions (improvement, social, conformity, and coping) [22,23]. Another questionnaire used for evaluation is the AUDIT, a self-assessment questionnaire for alcohol consumption, proposed by the World Health Organization (WHO). It has a total of 10 items and identifies risky consumption and possible dependence [24,25,26]. The CAGE questionnaire, a short four-item instrument, evaluates the possibility that there is a problem with consumption [27].

Although previous research has a broad approach to both the reasons and the risk of alcohol consumption in the adult population, the instruments for evaluating the motivations for consumption do not consider a classification of severity of consumption, particularly in university students, which would allow an early risk evaluation. Also, the existing instrument designs have yet to consider specific cultural and demographic differences for the student population. Therefore, this study aimed to design and validate a quantitative scale of motivations that evaluates moderate and severe alcohol consumption in Mexican university students. We present the designed scale and its validation by exploratory and confirmatory analyses that revealed a three-factor composition for moderate consumption and a four-factor composition for severe consumption. Moreover, we show how some of these factors are associated with the risk perception of alcohol and other substance consumption. This can depend on the level of consumption, particularly with the motivation of alcohol use as a social mediator. We further discuss how the new instrument could be used to develop effective strategies and support actions aimed at decreasing alcohol consumption and increasing the students’ well-being and mental health.

## 2. Materials and Methods

### 2.1. Stage 1: Design and Construction of the Instrument

#### 2.1.1. Participants

Twenty undergraduate students were non-randomly selected and asked about their primary motivations for drinking alcohol in the past year. Their 27 motivations were subsequently used to conduct a pilot survey of exploratory data in a convenience sample of 130 university students from three undergraduate programs in a public University of Orizaba, Veracruz, Mexico, aged between 18 and 24, and of both sexes (82 women, 48 men). The methods used for the instrument’s design are highly qualitative (i.e., the aim is to identify the words or items with more substantial semantic weight). Therefore, a random sample of the participants is not required.

#### 2.1.2. Instrument

**Figure 1:**
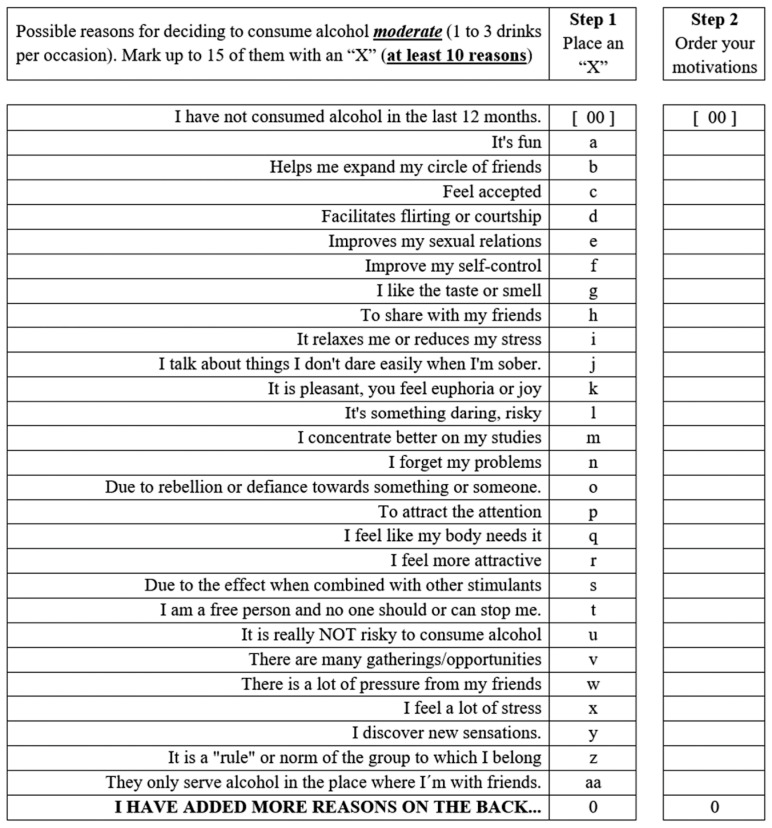
English translation of the form used to design the instrument at a moderate level. The word severe was used in an additional form for students who reported consuming four or more drinks per occasion.

Three dimensions of motivations for alcohol consumption were defined: (i) Low-risk perception or defiant behavior (i.e., to assume that alcohol consumption is harmless and without detrimental effects, that it can be performed without limits or consequences); (ii) Mediator or social disinhibitor (i.e., use alcohol as a resource to establish or facilitate social contact, freedom, or achieve desirable goals); and (iii) Somatic-sensory beneficial effects of alcohol (i.e., to attribute beneficial or pleasurable effects to alcohol). A questionnaire was designed with a stimulus sentence: “Possible motivations for deciding to consume alcohol moderately (1 to 3 drinks per occasion)” or “Possible motivations for deciding to consume alcohol at a severe consumption level (4 or more drinks per occasion)”. Twenty-seven sentences were summarized in a format where the sentence was the same for both consumption levels but differed, respectively, in the last word: moderately (1 to 3 drinks per occasion) or severe consumption (4 or more drinks per occasion) (Figure 1).

#### 2.1.3. Procedure

Once the objective of the study was explained, and students were invited to participate voluntarily, each student signed an informed consent form following local and international ethical guidelines. Then, the questionnaires were administered individually following the procedures proposed by [28]. Furthermore, the team that administered the questionnaires underwent prior training to ensure neutrality in the questions and to minimize bias in the students’ responses. Informed consent was obtained, highlighting the importance of confidentiality and anonymity. The items were evaluated by a group of professional experts to ensure that the questions did not contain elements that would lead to suggestive responses and to mitigate social desirability. Each student was given a list of 27 motivations for consuming alcohol and asked to select all the reasons or motivations they had used to drink alcohol, leaving them open to the possibility of adding others (Figure 1). They were asked to order each of the indicated motivations by assigning the number one to the one that best defined the most potent reason, followed by number two, until they had finished all the motivations they had chosen or added. When participants reported that they had drunk both moderately and severely, they were asked to fill out both formats.

To identify the network core (main words or components derived from the words recognized as most frequent), we followed the procedure recommended by Reyes (1993) and Mercado et al. (2015) [28,29]. Once participants wrote a word in response to the stimulus or question, they were asked to ‘rate’ it from 1 to 10, with 1 representing the highest semantic significance according to the word they had written. In this way, each word had a specific numerical value assigned by each participant. Later, this rating is inverted (i.e., the number 1 is assigned as number 10, the number 2 is assigned as number 9, and so on). Then, the numerical rating of words mentioned by participants was multiplied by the number of times they were mentioned to obtain an adjusted sum (semantic weight). The words with the highest semantic weight were ordered from highest to lowest and plotted to visually identify the breaking point, where the line becomes asymptotic to the horizontal axis (i.e., Catell’s breaking point) [30]. All the words with the highest scores were retained in the pilot instrument, thus creating the network core, with the words with the lowest numerical strength being eliminated.

### 2.2. Stage 2: Validity and Reliability of the Instrument

#### 2.2.1. Participants

A stratified probabilistic sample of 402 students between the ages of 19 and 22 of both sexes (262 women, 140 men) was obtained from five different undergraduate programs at the Universidad Veracruzana, Campus Ixtaczoquitlán, Veracruz, Mexico (Table 1). From the official list of 1609 active students, participants were randomly selected to include 25% of students of each academic program offered on the campus.

#### 2.2.2. Instrument

The motivational reasons for drinking alcohol that emerged in the first stage were used to integrate an instrument for evaluating motivations for alcohol consumption in university students. The perceived risk of alcohol consumption was also assessed based on item 45 from the 2009 Student Survey [31]. Item 45 is valid and reliable for evaluating the perception of risk to evaluate/assess the risk of consuming various substances in the Mexican student population. It consists of a closed question that is answered with three options ranging from 1 (it is not dangerous) to 3 (it is very dangerous).

#### 2.2.3. Procedure

The study’s objective was explained to each selected student, and they were invited to participate voluntarily. Once they accepted, they were asked to fill out and sign an informed consent form. They then proceeded to fill out all the items individually. The questionnaire was applied in small groups or individually. The student was given a copy of the test, the printed instructions were read and explained, a test exercise was performed to make sure they understood the instructions, a time slot was given to answer doubts, and they proceeded to fill out the questionnaire, which lasted between 5 and 10 min in total.

#### 2.2.4. Statistical Analysis

The reliability analysis was carried out in several steps: First, the distribution was analyzed, item by item, identifying if there were out of range values, and that all response options had at least five choices or answers. Then, the bias of each item was observed and analyzed, looking for a non-central tendency of the data (university population with alcoholism, that is, non-typical students), with a range between −1.5 and +1.5: from the total score for each item, the scores of the extreme quartiles were identified. We continued by creating dummy variables, called “extremes”, with quartiles 1 (25%) and 3 (75%), which belong to the lower and upper groups of all participants, thus identifying two groups: with low scores and with high scores. Next, a t-test for independent samples was carried out with the total scores of both groups to identify the discriminative capacity of the extreme quartiles. Furthermore, to verify the directionality of the reagents, an analysis was performed using cross tables, placing the extremes in rows and the reagents in columns. Then, a correlation assessment was carried out between all the items to identify their magnitude, directionality, and significance (for the subsequent decision on the type of factor analysis). Finally, the Cronbach’s alpha reliability was calculated, as well as the correlation of each item against the other items and the correlation of each item with the total, verifying that no item had a Cronbach’s alpha greater than the total (increased alpha) when the item was removed from the instrument.

To validate the instrument and estimate its factorial structure, an exploratory analysis was carried out using the Varimax rotation method (because the correlations between all the items tended to be at low to medium values). The analysis criteria included the following: (i) minimum factor loadings of 0.40, (ii) a minimum of two items per factor were expected, and (iii) the minimum expected intraclass correlation coefficient was 0.700, due to the interval numerical scale of the items, with an alpha error of *p* ≤ 0.05, (iv) The cut-off value for a minimum loading factor of 0.40, irrespective of sample size, has been suggested by Stevens (1992) and is helpful for interpretative purposes [32]. The outgoing rotated component matrix of the varimax analysis was analyzed to identify reagents that appeared in more than one factor (which would cause it to be eliminated from the instrument, due to its uncertainty and lack of specific location, which would mean that it was not valid to measure a given factor, but rather two or more and could not be valid to be used clinically): (v) the usefulness of the structure of the components was determined using Bartlett’s test of sphericity (*p* ≤ 0.05) and the Keiser–Meyer–Olkin sampling adequacy index), (vi) the outgoing model with eigenvalues above 1.00, (vii) the percentage of explained variation in the model variance was observed, (viii) the necessary adjustments were made to the model if required.

Subsequently, with the factor structure from the previous exploratory analysis, a confirmatory factor analysis (CFA) was carried out using the maximum likelihood (ML) technique, which assumes a multifactor model with latent factors and estimates measurement errors or covariance between errors. The following steps were followed: (i) identification and specification of the model; (ii) estimation of standardized parameters (R2, correlations, modification indices, critical proportions of differences and covariances); (iii) the fit of the model was calculated, monitoring acceptable limits of its estimators, as well as non-collinearity between the measured variables. To estimate the structural equations of the model, we followed what was indicated by [33]. The AMOS 23 statistical program (IBM SPSS, Chicago, IL, USA) was used [34]. Several indices were estimated: X2 (CMIN), and the ratio between X2/degrees of freedom (CMIN/df) as a measure of model parsimony, the goodness-of-fit index (GFI) and its complements, the adjusted goodness index and the Tucker–Lewis Index (TLI), as well as the Comparative of Fit Index (CFI), which is considered the best indicator for samples around or greater than 200 [31,35]. Finally, the Root Mean Square Error of Approximation (RMSEA) indicates the model’s approximation to zero error.

## 3. Results

### 3.1. Stage 1: Design and Construction of the Instrument

Two subscales of motivation to drink alcohol with a motivational statement were designed: one for the moderate consumption level (students consuming one to three drinks per occasion) and another for the severe consumption level (consuming four or more drinks per occasion). The scale for moderate consumption obtained 34 motivational reasons to evaluate (network size), and the scale for severe consumption obtained 27 motivational reasons.

Of the total number of defining reasons for the stimulus phrase, only those that were in the core of the network (motivating reasons with the highest weighted frequency) and with the highest semantic weight (absolute sum of values according to the place assigned by the student to each motivating reason) were selected. The prototype instrument had 15 motivational reasons for each subscale (Table 2). Although the selected items were the same in both subscales, the order of the items was different, indicating a distinct relevance or importance of each motivation according to the consumption level. For moderate consumption, the motivations were low-risk perception or defiant behavior (n = 3), social mediator/disinhibitor (n = 5), and beneficial somatic-sensory effects of alcohol (n = 7). For severe consumption, the motivations were low-risk perception/ defiant behavior (n = 2), social mediator/ disinhibitor (n = 7), and somatic-sensory beneficial effects of alcohol (n = 6).

### 3.2. Stage 2: Validity and Reliability of the Instrument

When comparing the discrimination capacity of the items in the two subscales at both extremes (using Student’s *t*-test), the 15 items of each showed discriminative capacity. With the 15 items of each subscale that discriminated, directionality was evaluated using crosstabs, which led to the internal reliability test of each subscale through Cronbach’s alpha. After the reliability analysis, all items were retained because none were higher than the total alpha of each subscale. The correlational analysis between items yielded low and medium magnitudes (Supplementary Materials, Tables S1 and S2). Therefore, we performed principal components factor analysis.

An orthogonal factor analysis of principal components under the Varimax method was applied with the 15 items of each subscale. Starting from the sedimentation plot and the salient rotated components matrix, a structure with factor loadings greater than or equal to 0.40 and eigenvalues greater than one was obtained, as follows: for the moderate and severe consumption subscale, two and three factors, respectively. The sample adequacy indices (KMO = 0.849 for moderate consumption, KMO = 0.804 for severe consumption) for the factor analysis determined the usefulness of the structure of the salient components, and Bartlett’s sphericity tests indicated the non-identity of the respective correlation matrices.

By eliminating duplicate items in two or more factors, ten final items remained in the moderate consumption subscale (Table 3) and thirteen in the severe consumption subscale (Table 3), with a final total alpha of 0.886 and 0.884, respectively, explaining 57.5% and 70.5% of their variances. In the final subscales of the questionnaire for moderate consumption, items were confirmed for the three factors corresponding to the three motivational dimensions defined initially (Table 3): (i) Low-risk perception or defiant behavior (Factor 3 with two items), (ii) Social mediator or disinhibitory (Factor 2 with four items), and (iii) Beneficial somatic-sensory effects of alcohol (Factor 1 with four items).

In the final subscales of the questionnaire for severe consumption, items were confirmed for the three factors corresponding to the three motivational dimensions defined initially (Table 4): (i) Low-risk perception or defiant behavior (Factor 4 with two items), (ii) Mediator or social disinhibitor (Factor 2 with five items), and (iii) Beneficial somatic-sensory effects of alcohol (Factor 1 with four items). Additionally, the analysis identified a new dimension of evasion or avoidance (Factor 3 with two items).

Based on Spearman correlations, a positive and statistically significant relationship was found between the dimensions of both subscales (moderate and severe consumption), with each other, and with the risk for alcohol consumption (Table 5). The severe consumption subscale correlated with the risk of consuming other substances, such as marijuana, cocaine, inhalants, and cigarettes. The level of alcohol consumption obtained negative and statistically significant correlations with the consumption of alcohol, marijuana, cocaine, inhalants, and cigarettes.

Based on the exploratory analysis of each of the instruments (moderate and severe consumption), the adjustment of the factors of the previous exploratory analysis was evaluated using confirmatory factor analysis (CFA), using the maximum likelihood method, which included the steps of model identification and specification, the estimation of standardized parameters (correlations R^2^, covariances, modification indexes, and critical difference ratios). Finally, the fit was evaluated by observing the acceptable limits of the estimators, as well as non-collinearity in the measured variables. The global fit indices X2 and the best ratio indicator for samples equal to or greater than 200, and finally, the root mean square error of approximation (RMSEA) [22,31,36,37] were evaluated.

**Figure 2:**
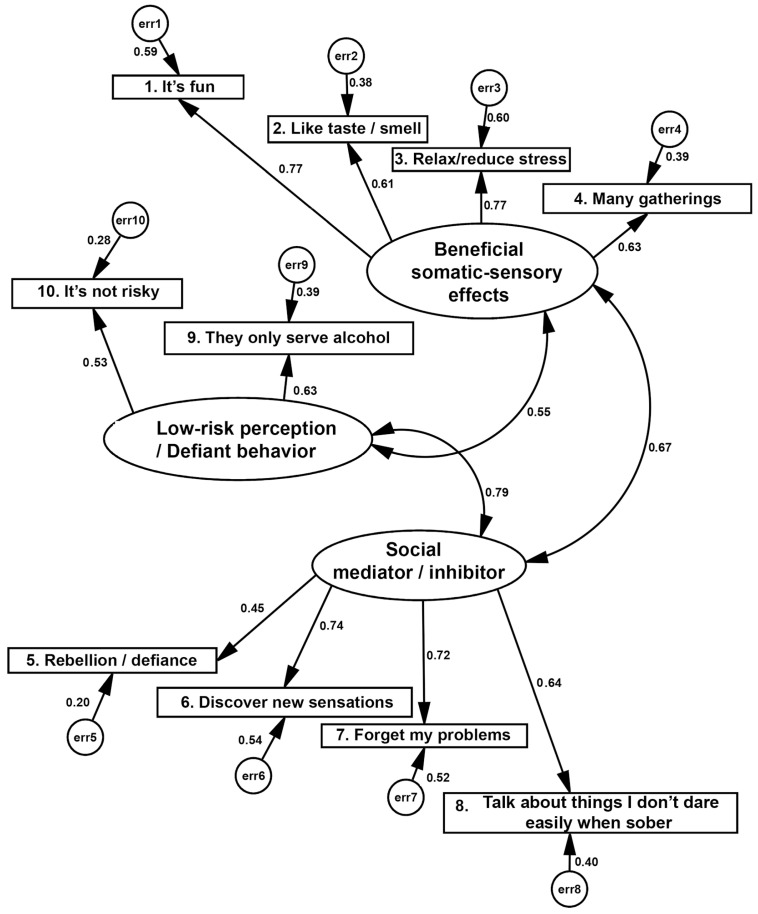
Confirmatory model of three factors of motivations for moderate alcohol consumption in Mexican university students (n = 225). Chi-squared = 64.93, 32 df, *p* ≤ 0.001; CMIN/df = 2.03; RMR = 0.055; GFI = 0.946; CFI = 0.947; RMSEA = 0.068 (0.044–0.091).

For moderate consumption, the chi-square/gl ratio of overall fit (64.9/32 gl = 2.03, *p* = 0.001) indicated near-zero errors in the variances and covariances of the model fitted to this population (Figure 2). The main goodness-of-fit index (GFI = 0.946) and the comparative index (CFI = 0.947) confirm the model in Figure 2 as complex and acceptable, while the index RMR = 0.055 (close to 0) and RMSEA = 0.088 confirm it since they penalize the complexity. Still, these indices remain below or very close to 0.05 and 0.08, respectively, so the model is recursive and is correctly identified. The factor loadings and the error variance are indicated for each item.

**Figure 3:**
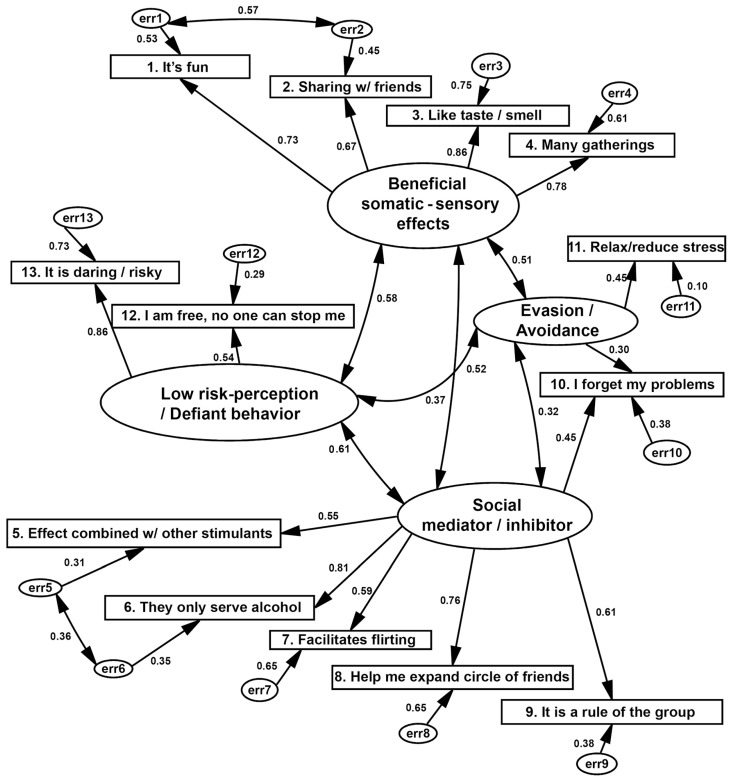
Confirmatory model of four factors of motivations for severe alcohol consumption in Mexican university students (n = 82). Chi-squared = 80.61, 56 df, *p* ≤ 0.001; CMIN/df = 1.44; RMR = 0.091; GFI = 0.877; CFI = 0.949; RMSEA = 0.074 (0.032–0.108).

For severe consumption, the chi-square/df ratio of global fit (80.61/56 df = 1.44, *p* = 0.001) indicated almost zero errors in the variances and covariances of the model adjusted to this population (Figure 3). The main goodness-of-fit index (GFI = 0.877) and the comparative index (CFI = 0.949) confirm the model as complex and acceptable, while RMR = 0.091 (close to 0) and RMSEA = 0.074 confirm it since they penalize the complexity. Still, the indices remain below or very close to 0.05 and 0.08, respectively, so the model is recursive and is correctly identified.

## 4. Discussion

### 4.1. Main Contribution

Evaluating alcohol consumption in university students is based on research using traditional questionnaires [38]. This approach does not consider individual characteristics and particular elements such as motivations, which it assumes could be a factor or predictor of alcohol consumption [34,35]. The psychometric data produced by this study demonstrate that the proposed instruments are reliable and valid measures for evaluating the motivations that lead to alcohol consumption among university students.

### 4.2. Comparison with Previous Scales of Alcohol-Drinking Motivations

The results of the study show the importance of the classification of motivations. The instrument’s prototype (Table 2) showed that the main motivations were low-risk perception, social disinhibition, and sensory somatic effects. This coincides with some studies that reinforce the same factors for alcohol consumption [3,17]. In the design stage, three dimensions of motivations for alcohol consumption were defined: low-risk perception, social mediator or disinhibitory, and beneficial sensory somatic effects of alcohol. This grouping coincides with previous reports [39,40]. In the second phase of the study, the exploratory analysis showed that the motivations are related to the level of consumption; at the moderate level, there are three dimensions (low perception of risk, social disinhibitory, and somatic effects), while in severe consumption, there were four because the evasion dimension was added. Some studies coincide with what was reported in this study, since they refer to the difficulty in perceiving risk [38,39], the social connotations it has [41], and the role that alcohol has as an evader in complex life situations [3,17]. The dimensions identified in the present questionnaire align with theoretical models such as expectancy theory [42], social learning theory [43], and the theory of planned behavior [44], which include avoidance behaviors and low-risk perception among the factors that explain excessive alcohol consumption.

The severe consumption subscale shows correlations with the risk perception for consumption of other substances, such as marijuana, cocaine, inhalants, or cigarettes (Table 5). This coincides with studies that relate high alcohol consumption with the possibility of addiction [41,45,46]. The relationship between severe alcohol consumption and the perception of low risk shown in the study exposes the threat of normalization and an increase in consumption among university students. A perception of invulnerability can lead to erroneous perceptions, such as the benevolent conception of alcohol and the cognitive association in an alcohol–fun binomial, which leads to severe consumption behaviors [47]. In contrast, moderate consumption does not correlate with the risk perception for consumption of other substances (Table 5). The lack of correlation suggests that the consumption of other substances may not be a factor involved in moderate alcohol consumption. Although this could seem contradictory to alcohol use as a predictor of consumption of other drugs [4], the discrepancy may be explained by the analysis based on dichotomized variables (use of alcohol and use of drugs categorized as yes or no) without considering the level of alcohol consumption. Hence, it is likely that such an association could be attributed mainly to heavy drinkers. This highlights the relevance of investigating alcohol use and motivations for alcohol use with explicit assessment of the level of consumption.

Regarding the psychometric properties, the questionnaire proposed in the present work showed excellent levels of reliability (Cronbach’s alpha) in assessing motivations for moderate and severe alcohol consumption: 0.886 and 0.884, respectively. Moreover, the confirmatory analysis showed adequate goodness-of-fit indices, for instance, CFI of 0.947 and 0.949, respectively, and RMSEA of 0.068 and 0.074, respectively. Other questionnaires that assess alcohol consumption in Mexican or Latin-American students showed equivalent indices: CAGE had a Cronbach’s alpha of 0.73 [48,49], AUDIT had a Cronbach’s alpha of 0.75 [50], and DMQR had a Cronbach’s alpha of 0.86, CFI of 0.912, and RMSEA of 0.081 [51]. Noticeably, the present questionnaire showed very good psychometric properties on the confirmatory analysis, while most studies in this population lacked confirmatory analysis.

### 4.3. Relevance of Motives for Alcohol Consumption in the Relationship Between Drinking Alcohol, Mental Health, and Well-Being

Alcohol consumption impacts public health from different dimensions. There is clear evidence of a link between alcohol misuse and mental health and well-being [17,52]. The term mental well-being denotes positivity in the concept, referring to the mental state of an individual, how they are feeling, and their ability to cope with day-to-day stressors, regardless of any diagnostic condition or mental pathology [53]. Hazardous alcohol intake is associated with low mental well-being [16,54,55]. Alcohol consumption has a comorbidity between harmful drinking and diagnosed mental illnesses, such as depression and anxiety [52,56]. This denotes a risk for the increase in mental pathologies. A negative correlation has been demonstrated between well-being and alcohol consumption and states of happiness. An increase in alcohol consumption decreases the perception of well-being, pleasure, and happiness, which is associated with poor mental well-being [12].

In this study, some motivators that contribute to alcohol consumption were identified, including low-risk perception, the use of drinking as a social mediator, and a momentary sensory effect. These motivators could affect the students’ well-being level, for instance, through the negative relationships between alcohol consumption and low-risk perception [55,57,58]. When a student does not appreciate the danger of drinking alcohol, consumption increases, as well as the subsequent risk of adverse consequences. They may experience psychological discomfort associated with guilt or sadness [12,13,46,53,59]. Another negative consequence of the motivators found with mental health is that alcohol consumption can generate a feeling of well-being and security that results in placebo effects and that, ultimately, leads to damage to mental health.

### 4.4. Clinical Implications for Mental Health and Well-Being in College Students

The questionnaire develops the possibility of a further understanding of the psychological mechanisms of this relationship in the Mexican population, in addition to the contribution it represents in the spheres of prevention of alcohol abuse and promotion of our student’s well-being [60]. Among the many approaches for college drinking interventions, one relevant example for promoting well-being is protective behavioral strategies (PBS), which are based on self-determination theory and can minimize the harm associated with alcohol use [55]. The use of PBS mediates the effect of certain motivations on alcohol use (social and enhancement motivations) but not the effects on coping motivations (i.e., seeking the amelioration of one’s negative mood). Since there are unique relations between drinking motives and the motivation for drinking responsibly (for instance, by using PBS), assessing motivation is important for a better understanding of alcohol-related behaviors and more effective college drinking interventions [1]. Moreover, the mediating role of drinking motives and PBS is particularly relevant in those students with more depressive symptoms, who could benefit from clinical interventions tailored to address negative reinforcement motives, increase PBS use, and minimize harm [61].

Another example in which identifying the motives for alcohol consumption is relevant for effective treatment among college students is in those with social anxiety or who do not fit in on campus, which is associated with more significant amounts of drinks per week [57] and binge drinking [58]. Improving interventions for those students requires identifying such motives and implementing measures to increase their perception of belonging or fitting in [57,58]. Also, it has been proposed that strategies to strengthen resilience among Hispanic college students, whose drinking is motivated by the desire for socialization, could be protective against alcohol-related consequences [60]. The strong psychometric properties demonstrated in the proposed questionnaire support the promising use of this tool in Mexican university students. For instance, the identification of motives for severe drinking may be useful to develop interventions aimed at tackling alcohol abuse in this population.

### 4.5. Study Limitations

The study was carried out on a sample of students in a region of Mexico, and future studies are required with larger and more heterogeneous samples across Mexico to increase the generalization of the results. Further studies are needed to identify the influence of gender and other factors on the motivations for alcohol consumption, both moderate and severe. To extend the concurrent external validity of the instrument proposed in this study, it is necessary to contrast the developed instrument with other known motivation questionnaires, such as the DAQAR, and to correlate the motivations with the consumption level (e.g., AUDIT or CAGE). The validation of the subscale for severe drinking was relatively small (n = 82), increasing the risk of overfitting the models of the confirmatory analysis. Further studies with larger samples are needed to confirm the good model fit obtained in the present study.

## 5. Conclusions

This study presents an instrument to evaluate motivations for consuming alcohol among Mexican university students, which includes motivations for moderate consumption (with three dimensions) and for severe consumption (with four dimensions). Through exploratory and confirmatory analysis, it was demonstrated that the proposed instrument is reliable and valid. Moreover, a correlation analysis showed that the dimensions of the developed instrument are associated with the perception of the risk of ingesting alcohol and other substances. Having validated instruments specific to this university student population presents an invaluable opportunity that can complement and support preventive work and timely intervention to prevent or alleviate mental health issues and increase well-being in this population. Furthermore, this new instrument has the potential of broader applicability in designing culturally specific prevention and intervention programs. It could also be adapted for other populations or regions with similar cultural contexts.

## Figures and Tables

**Table 1:** Characteristics of the probabilistic sample of students.

		Alcohol Consumption Level			
Variable	Total (N = 402)	None (N = 95)	Moderate (N = 225)	Severe (N = 82)	*p*-Value
Academic program					0.175
Accounting	125 (31%)	32 (34%)	76 (34%)	17 (21%)	
Administration	113 (28%)	22 (23%)	66 (29%)	25 (32%)	
Computational Systems	66 (16%)	13 (14%)	32 (14%)	21 (26%)	
Bussines management	66 (16%)	18 (19%)	35 (16%)	13 (16%)	
Informatics	32 (8%)	10 (10%)	16 (7%)	6 (7%)	
Gender					0.001
Female	262 (65%)	70 (74%)	152 (68%)	40 (49%)	
Male	140 (35%)	25 (26%)	73 (32%)	42 (51%)	

**Table 2:** Motivational reasons for alcohol consumption were selected during the design and sorted by their semantic weight (SW).

Moderate Consumption (1 to 3 Drinks per Occasion) N = 225	SW	Severe Consumption (4 or More Drinks per Occasion) N = 82	SW
To share with my friends	731	To share with my friends	236
I like the taste or smell	478	It’s fun	207
It’s fun	411	I like the taste or smell	207
It relaxes me and reduces my stress	350	It relaxes me and reduces my stress	172
There are many gatherings/opportunities	283	There are many gatherings/opportunities	147
It is pleasant. You feel euphoria or joy	161	It is pleasant. You feel euphoria or joy	109
I forget my problems	150	I forget my problems	107
They only serve alcohol in the place where I’m with friends	135	It helps me expand my circle of friends	74
It helps me expand my circle of friends	108	Facilitates flirting or courtship.	69
I talk about things I don’t dare easily when I’m sober.	101	It is a “rule” or norm of the group to which I belong	60
It is NOT risky to consume alcohol	98	They only serve alcohol in the place where I’m with friends	40
I am a free person, and no one should or can stop me	91	Due to the effect when combined with other stimulants	36
Facilitates flirting or courtship	77	It’s something daring, risky	31
I discover new sensations	68	I feel a lot of stress	28
Out of rebellion or defiance towards something or someone	55	I am a free person, and no one should or can stop me	27

Stimulus phrase: “Possible reasons for deciding to consume alcohol …” (Moderately or severely depending on the reported consumption).

**Table 3:** Distribution of reagents for moderate consumption (N = 225). Results of the factor analysis with orthogonal rotation, with total variance explained by the scale = 57.5%. The numbers represent factor loading. Factor 1 = Beneficial somatic-sensory effects; factor 2 = Social Mediator/Inhibitor; factor 3 = Low-risk perception/defiant behavior.

Items	Factor			Mean ± SD
	1	2	3	
It’s fun I like the taste or smell It relaxes me or reduces my stress There are many gatherings/opportunities	0.783 0.766 0.729 0.647			3.1 ± 1.2 3.2 ± 1.2 3.6 ± 1.2 3.2 ± 1.2
5. Due to rebellion or defiance towards something or someone 6. I discover new sensations 7. I forget my problems 8. I talk about things I don’t dare easily when sober		0.765 0.739 0.654 0.485		4.7 ± 0.7 4.3 ± 0.9 4.3 ± 1.0 4.2 ± 1.0
9. They only serve alcohol in the place where I’m with friends 10. It is not risky to consume alcohol			0.740 0.698	3.7 ± 1.2 3.7 ± 1.3
Cronbach’s alpha of the factor	0.791	0.730	0.498	
Percentage of variance explained	39.6%	11.1%	6.8%	
Arithmetic average	13.0	17.6	7.4	
Standard deviation	3.7	2.8	2.0	
Factor variance	13.4	7.6	4.2	
Intraclass factor correlation	0.487	0.404	0.332	
Lower correlation value	0.420	0.335	0.210	
Higher correlation value	0.554	0.475	0.443	
F value	4.8	3.7	2.0	
*p*-value	≤0.001	≤0.001	≤0.001	

**Table 4:** Distribution of reagents for severe consumption (N = 82). Results of the factor analysis with orthogonal rotation, with total variance explained by the scale = 70.5%. The numbers represent factor loading. Factor 1 = Beneficial somatic-sensory effects; factor 2 = Social Mediator/Inhibitor; factor 3 = Evasion/Avoidance; factor 4 = Low-risk perception or defiant behavior.

Items	Factor				Mean ± SD
	1	2	3	4	
It’s fun	0.846				2.7 ± 1.4
2. For sharing with my friends	0.799				2.3 ± 1.4
3. I like the taste or smell	0.774				2.9 ± 1.3
4. There are many gatherings/opportunities	0.701				3.0 ± 1.3
5. Due to the effect when combined with other stimulants		0.858			4.6 ± 0.9
6. They only serve alcohol in the place where I’m with friends		0.825			3.8 ± 1.3
7. Facilitates flirting or courtship.		0.684			3.9 ± 1.3
8. Helps me expand my circle of friends		0.596			3.7 ± 1.2
9. It is a “rule” or norm of the group to which I belong		0.588			4.3 ± 1.1
10. I forget my problems			0.892		3.9 ± 1.2
11. It relaxes me or reduces my stress			0.697		3.3 ± 1.3
12. I am free, and no one should or can stop me				0.929	3.8 ± 1.3
13. It is something daring, risky				0.623	3.9 ± 1.2
Cronbach’s alpha of the factor	0.876	0.810	0.777	0.629	
Percentage of variance explained	43.0%	12.7%	7.6%	7.2%	
Arithmetic average	11.0	20.2	7.2	7.7	
Standard deviation	4.6	4.4	2.3	2.2	
Factor variance	21.2	19.3	5.4	4.8	
Intraclass factor correlation	0.638	0.459	0.636	0.459	
Lower correlation value	0.542	0.357	0.486	0.270	
Higher correlation value	0.728	0.567	0.749	0.614	
F value	8.1	5.3	4.5	2.7	
*p*-value	≤0.001	≤0.001	≤0.001	≤0.001	

**Table 5:** Spearman correlation coefficients between risk perception for alcohol and other substance consumption vs. level of alcohol consumption and motivations for alcohol consumption in university students.

	How Dangerous (for Anyone’s Health) Do You Consider It to Be…?					
	Consume Alcohol	Consume Marijuana	Consume Heroin	Consume Cocaine	Consume Inhalants	Smoking Five or More Cigarettes per Day
Risk perception for alcohol consumption (N = 402)	1.000	0.267 **	0.202 **	0.218 **	0.265 **	0.372 **
Alcohol consumption level (N = 402)	−0.193 **	−0.153 ***	−0.042	−0.109 *	−0.125 *	−0.214 ***
Motivations to consume alcohol						
Moderate consumption (N = 225)						
Factor 1 (Beneficial somatic-sensory effects)	0.218 **	0.100	0.015	0.015	−0.043	−0.043
Factor 2 (Social mediator/disinhibitor)	0.278 **	0.095	0.106	0.095	0.006	0.027
Factor 3 (Low-risk perception/Defiant behavior)	0.192 **	0.045	0.065	0.032	−0.018	−0.005
Severe consumption (N = 82)						
Factor 1 (Beneficial somatic-sensory effects)	0.296 **	0.270 *	0.014	0.023	0.081	0.100
Factor 2 (Social mediator/disinhibitor)	0.293 **	0.166	0.258 **	0.236 *	0.281 **	0.311 **
Factor 3 (Evasion)	0.372 **	0.199 *	0.110	0.143	0.107	0.240 *
Factor 4 (Low perception of risk or defiant behavior)	0.246 *	0.087	0.028	0.019	0.059	0.082

* *p* < 0.05, ** *p* < 0.01, *** *p* < 0.001.

## Data Availability

The authors will make the raw data supporting this article’s conclusions available upon request.

## Supplementary Materials

The following supporting information can be downloaded at: https://www.mdpi.com/article/10.3390/healthcare13030307/s1, Table S1: Inter-item Spearman correlations of the moderate consumption questionnaire (N = 225); Table S2: Inter-item Spearman correlations of the severe consumption questionnaire (N = 82).
