# Supplementary material for: Validation of a Questionnaire of Motivations for Moderated and Severe Alcohol Consumption Among College Students
Source: Healthcare (Basel). 2025 Feb 2;13(3):307. doi: 10.3390/healthcare13030307 (PMC11817057; doi:10.3390/healthcare13030307)
Supplement: Supplementary file 1 [file healthcare-13-00307-s001.zip › SupplementaryMaterial.pdf]

Supplementary material

Table S1. Inter-item Spearman correlations of the **moderate** consumption questionnaire (N = 225).

**Items**	I like the taste or smell	It´s fun	It relaxes me or reduces my stress	There are many gatherings/opportunities	I forget my problems	They only serve alcohol in the place where I´m with friends	I talk about things I don't dare easily when sober	It is really NOT risky to consume alcohol	I discover new sensations	Due to rebellion or defiance towards something or someone
I like the taste or smell										
It´s fun	0.492**									
It relaxes me or reduces my stress	0.495**	0.569**								
There are many gatherings/opportunities	0.394**	0.480**	0.422**							
I forget my problems	0.195**	0.375**	0.526**	0.300**						
They only serve alcohol in the place where I´m with friends	0.166*	0.290**	0.220**	0.246**	0.333**					
I talk about things I don't dare easily when sober	0.223**	0.347**	0.326**	0.310**	0.414**	0.324**				
It is really NOT risky to consume alcohol	0.163*	0.280**	0.214**	0.191**	0.224**	0.346**	0.386**			
I discover new sensations	0.176**	0.296**	0.341**	0.289**	0.496**	0.395**	0.498**	0.374**		
Due to rebellion or defiance towards something or someone	0.006	0.122	0.200**	0.117	0.389**	0.148*	0.303**	0.168*	0.484**	

** The correlation is significant at the 0.01 level (two-tailed).

* The correlation is significant at the 0.05 level (two-tailed).

Table S2. Inter-item Spearman correlations of the **severe** consumption questionnaire (N = 82).

**Items**	It´s fun	For sharing with my friends	I like the taste or smell	There are many gatherings/opportunities	Due to the effect when combined with other stimulants	They only serve alcohol in the place where I´m with friends.	Facilitates flirting or courtship.	Helps me expand my circle of friends	It is a "rule" or norm of the group to which I belong	I forget my problems	It relaxes me or reduces my stress	I am free, and no one should or can stop me	It is something daring, risky
It´s fun													
For sharing with my friends	0.746**												
I like the taste or smell	0.575**	0.515**											
There are many gatherings/opportunities	0.571**	0.548**	0.604**										
Due to the effect when combined with other stimulants	0.050	0.033	0.139	0.245*									
They only serve alcohol in the place where I´m with friends	0.228*	0.275*	0.330**	0.318**	0.534**								
Facilitates flirting or courtship.	0.293**	0.427**	0.331**	0.301**	0.384**	0.500**							
Helps me expand my circle of friends	0.263*	0.338**	0.207	0.316**	0.381**	0.507**	0.659**						
It is a "rule" or norm of the group to which I belong	0.239*	0.349**	0.107	0.282*	0.502**	0.406**	0.467**	0.478**					
I forget my problems	0.348**	0.296**	0.308**	0.270*	0.283**	0.377**	0.512**	0.483**	0.494**				
It relaxes me or reduces my stress	0.434**	0.407**	0.694**	0.544**	0.186	0.383**	0.416**	0.315**	0.290**	0.612**			
I am free, and no one should or can stop me	0.251*	0.258*	0.175	0.401**	0.140	0.205	0.366**	0.350**	0.290**	0.207	0.313**		
It is something daring, risky	0.427**	0.313**	0.386**	0.460**	0.305**	0.457**	0.363**	0.420**	0.431**	0.365**	0.468**	0.503**	

** The correlation is significant at the 0.01 level (two-tailed).

* The correlation is significant at the 0.05 level (two-tailed).
